# Contralateral Fallopian Tube Dermoid Tumor: A Rare Finding in Chronic Tubal Ectopic Gestation

**DOI:** 10.7759/cureus.8065

**Published:** 2020-05-12

**Authors:** Sweta Singh, Prashanta K Rout, Rishila Majumder

**Affiliations:** 1 Obstetrics and Gynecology, All India Institute of Medical Sciences Bhubaneshwar, Bhubaneswar, IND; 2 Obstetrics and Gynecology, All India Institute of Medical Sciences Bhubaneswar, Bhubaneswar, IND; 3 Pathology and Laboratory Medicine, All India Institute of Medical Sciences Bhubaneswar, Bhubaneswar, IND

**Keywords:** chronic tubal ectopic gestation, fallopian tube dermoid, ultrasonography

## Abstract

Mature cystic teratoma is the most common benign tumor of the ovaries in women of reproductive age. Dermoid tumor in an ectopic or aberrant location is usually detected incidentally. The occurrence of a dermoid tumor in the fallopian tubes is extremely rare, with only approximately 75 cases being reported worldwide in the English language literature. This report describes a left-sided fallopian tube dermoid tumor in a 27-year-old woman with chronic right tubal ectopic gestation and discusses its clinical implications. Inspection of the contralateral fallopian tube in a woman with a ruptured tubal ectopic pregnancy is required clinically to rule out bilateral tubal ectopic gestation. Although rare, the ectopic presence of a dermoid tumor in the fallopian tube may be an incidental finding requiring surgical removal.

## Introduction

Mature cystic teratoma, also known as dermoid tumor or cyst, is the commonest benign tumor of the ovaries in women of reproductive age group, comprising 16% to 20% of all ovarian tumors [1]. Dermoid tumor in an ectopic or aberrant location is usually detected during cesarean delivery or laparoscopic sterilization [2]. Its occurrence in the fallopian tubes is extremely rare, with only approximately 75 cases having been reported worldwide [3]. This case report highlights the possibility of fallopian tube dermoid as an incidental finding in patients with chronic tubal ectopic gestation and that these tumors require surgical removal.

## Case presentation

A 27-year-old woman presented with a history of continuous but scant bleeding per vaginum for the previous two months following a self-managed abortion with tablet mifepristone and misoprostol at six weeks of gestation. Her previous menstrual cycles were regular with average flow. She had a full-term normal vaginal delivery three years earlier. There was no history of contraceptive usage, and both her previous and family history were unremarkable. On examination, she was conscious and oriented, with stable vital signs but a mild degree of pallor. Her abdomen was soft with no rigidity, guarding, tenderness, or palpable masses. The speculum examination revealed a normal cervix with no bleeding per vaginum. Bimanual examination showed that her uterus was bulky, retroverted, and soft, with side-to-side mobility and fullness in the pouch of Douglas (POD). Her urine pregnancy test was positive, her hemoglobin concentration was 8.3 g/dl, and her serum beta-human chorionic gonadotropin concentration was 654 mIU/ml.

**Figure 1:**
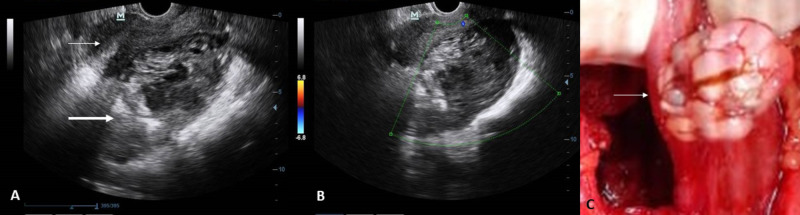
Ultrasound and intraoperative image (A) Two-dimensional ultrasonography showing a normal-sized uterus (thin arrow) with a well-defined heterogeneous lesion in the pouch of Douglas (POD) measuring 9.0 x 8.5 x 6.4 cm (thick arrow). (B) Color Doppler ultrasonography showing no internal vascularity in the lesion in the POD. (C) Intraoperative picture showing incidental finding of the left ovarian dermoid tumor (arrow).

Two-dimensional ultrasonography revealed a normal-sized uterus with a well-defined heterogeneous lesion in the POD measuring 9.0 x 8.5 x 6.4 cm (Figure 1A) with no internal vascularity on color Doppler (Figure 1B), suggestive of chronic ectopic gestation. On exploratory laparotomy, the POD contained approximately 100 ml of blood with clots. The right ovary and right fallopian tube, with dilatation and rupture over the ampullary region, were buried within the folds of the right broad ligament, with adhesions at the base of the rectosigmoid and covered with the omentum, forming an inflammatory mass measuring 10 x 8 cm. Careful adhesiolysis was performed, followed by right salpingectomy. Routine examination of the left fallopian tube showed a swelling within its ampullary region, which was firm to hard in consistency and measured 3 x 2 cm. Since bilateral tubal ectopic pregnancy was suspected, left salpingostomy was performed, resulting in the removal of an irregular white-colored solid lesion along with a few strands of hair, suggesting a primary left fallopian tube dermoid tumor (Figure 1C). She received one unit of packed cells postoperatively and was discharged uneventfully after four days.

**Figure 2:**
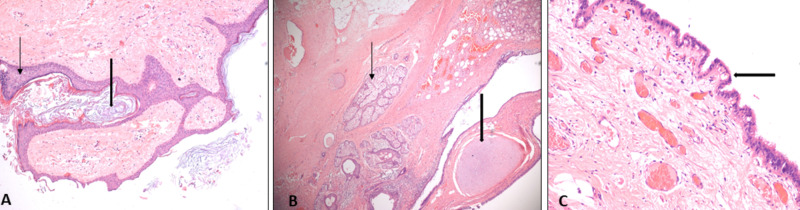
Histopathology image (A) Section showing epidermis (thin arrow) and keratin flakes (thick arrow). (B) Section showing skin adnexal structures (thin arrow) and cartilage (thick arrow). (C) Section showing respiratory epithelial lining (thick arrow).

Histopathology revealed that her right fallopian tube contained areas of intramural and transmural hemorrhage, trophoblastic cells, and occasionally degenerated chorionic villi, confirming a right tubal ectopic pregnancy. The solid lesion within the left fallopian tube showed the presence of epidermis with keratin flakes (Figure 2A), skin adnexal structures and cartilage (Figure 2B), and respiratory epithelium (Figure 2C), confirming the clinical diagnosis of a dermoid tumor of the left fallopian tube.

## Discussion

Dermoid tumors are common in the ovaries [1]. Their detection in ectopic or aberrant locations, including those in the POD, uterosacral ligaments, and round ligaments, is usually incidental during cesarean delivery or laparoscopic sterilization [2]. The location of dermoid tumors in the fallopian tubes is extremely rare, and is usually treated by surgery by laparoscopy or laparotomy [3]. Chronic tubal pregnancy, on the other hand, is a rare form of ectopic gestation, in which there is a gradual disintegration of the tubal wall with slow and/or repeated episodes of hemorrhage, leading to the formation of a pelvic mass in and around the adnexa and POD, resulting in adhesions and the formation of organized hematocele within the pelvis [4]. The treatment is salpingectomy as the fallopian tube is damaged.

A review of 30 patients with tubal dermoid tumors showed that four were associated with ectopic pregnancy, whereas none was associated with chronic tubal gestation on the contralateral side, as in our case [3]. This has clinical implications for future fertility potential, as it mandates a surgery on the contralateral tube after salpingectomy has been performed for chronic ectopic pregnancy on one tube. Fallopian tube dermoid tumors have been found in women from the age of 17 to 67 years, with a peak incidence during the third and fourth decades of life [1]. Our patient was 27 years old and was asymptomatic for the left fallopian tube dermoid tumor. It is possible that on ultrasound, the larger chronic tubal pregnancy on the right side masked the findings of the relatively smaller dermoid tumor of left fallopian tube in our case. Fallopian tube dermoid tumors have been reported to be associated with menstrual irregularity, reduced parity, abnormal discharge per vaginum, abdominal pain, postmenopausal bleeding, uterine fibroids, struma ovarii, uterine malformation, and endometrial carcinoma [3,5]. These tumors have been found to range in size from 0.5 to 17 cm (size increases with age); dermoid tumors grow slowly, at an average rate of 1.8 mm/year in premenopausal women [3].

Due to the rarity, the actual incidence and histogenesis of primary fallopian tube dermoid tumors remain unclear [1]. We performed right salpingectomy for the chronic tubal gestation, and opted for salpingostomy and removal of dermoid tumor of the left fallopian tube as a fertility preserving measure. Larger tumors have been misdiagnosed as those arising from the ovaries [3]. Treatment usually consists of surgical removal by laparoscopy or laparotomy, with these treatments being curative.

## Conclusions

Primary fallopian tube dermoid tumors are rare, and usually asymptomatic and diagnosed incidentally. If associated with a chronic tubal gestation on the contralateral side, management is challenging, as salpingectomy for the chronic tubal gestation needs to be combined with fertility preserving salpingostomy for surgical removal of the dermoid tumor of the contralateral fallopian tube. 
